# Comparative Analysis of Sleep Hygiene and Patterns among Adolescents in Two Russian Arctic Regions: A Pilot Study

**DOI:** 10.3390/children11030279

**Published:** 2024-02-24

**Authors:** Sergey N. Kolomeichuk, Lyudmila S. Korostovtseva, Artem V. Morozov, Michail V. Bochkarev, Yury V. Sviryaev, Dina A. Petrashova, Victoria V. Pozharskaya, Alexander A. Markov, Michail G. Poluektov, Denis G. Gubin

**Affiliations:** 1 Institute of Biology, Karelian Research Centre of the Russian Academy of Sciences, 185910 Petrozavodsk, Russia; artem.morozow@yandex.ru; 2 Group of Somnology, Almazov National Research Medical Center, 197341 Saint Petersburg, Russia; korostovtseva_ls@almazovcentre.ru (L.S.K.); sviryaev@almazovcentre.ru (Y.V.S.); 3 Laboratory for Genomics, Proteomics, and Metabolomics, Research Institute of Biomedicine and Biomedical Technologies, Tyumen State Medical University, 625023 Tyumen, Russia; alexdoktor@inbox.ru (A.A.M.); dgubin@mail.ru (D.G.G.); 4 Host LLC, 36 Engelsa Str., 620026 Ekaterinburg, Russia; dinapetrashova@mail.ru; 5 Federal Research Centre “Kola Science Centre of the Russian Academy of Sciences”, 184209 Apatity, Russia; v.pozharskaya@ksc.ru; 6 Department of Nervous Diseases, Sechenov Moscow Medical Institute, 119435 Moscow, Russia; poluektov_m_g@staff.sechenov.ru; 7 Department of Biology, Tyumen State Medical University, 625023 Tyumen, Russia; 8 Tyumen Cardiology Research Centre, Tomsk National Research Medical Center, Russian Academy of Science, 119991 Tyumen, Russia

**Keywords:** children, sleep hygiene, daytime sleepiness, sleep duration, Arctic

## Abstract

Purpose: The circumpolar habitat stands as one of the most vulnerable environments for human activity and health. The primary study objective was to compare sleep-related factors, light exposure, social cues, and potential confounding variables among schoolchildren residing in the European Arctic region from two settlements situated below and above the Polar Circle using validated self-reported questionnaires. Materials and Methods: We recruited 94 children aged 13–15 years (40.4% males), matched by sex and age, from public educational institutions in two circumpolar settlements located below (Kem’, Republic of Karelia; 64.6 NL) and above the Polar Circle (Apatity, Murmansk Region; 67.3 NL). Participants completed several surveys, including the Pediatric Daytime Sleepiness Scale, the Insomnia Severity Index, the Adolescent Sleep Hygiene Scale, and the Munich ChronoType Questionnaire, to evaluate sleep parameters and chronotype. The χ^2^ test was used to test for differences between proportions. Linear regression and multiple regression models with co-factors were applied to assess the relationship between studied indicators. Results: A noteworthy increase in physical activity was observed in children residing in Kem’ compared to those in Apatity. Children from Apatity showed higher alcohol consumption than their counterparts from Kem’. The overall rate of excessive daytime sleepiness in the sample was 17.1%. Moderate insomnia symptoms were reported in 18.4% of adolescents living in Kem’ and in 25% of respondents living in Apatity, respectively. Notably, participants from Kem’ attained higher academic scores and had longer exposure to sunlight on schooldays. On the other hand, children from Apatity tended to have later bedtimes and sleep-onset times on schooldays. According to the Munich ChronoType Questionnaire data, a reliance on alarm clocks on schooldays, and a higher Sleep Stability Factor based on the Adolescent Sleep Hygiene Scale. Discussion: Our study indicating that higher physical activity and longer sunlight exposure among Kem’ children on schooldays are associated with earlier wake-up times during schooldays, earlier bedtime whole week, reduced dependence on alarm clocks, and higher academic achievements. The results of older schoolchildren differ from many works published previously in the USA, Argentina, and Japan, which could be explained by the season when the study was performed. Here, we observed a negative impact on school performance and sleep parameters in children living in high latitudes, namely in circumpolar regions. Conclusions: Our study points out that adolescents living above the Polar Circle tend to have sleep problems, e.g., late sleep-onset times, higher excessive daytime sleepiness, and insomnia-related symptoms, because of experiencing reduced exposure to natural light. Future research encompassing assessments across all four seasons will provide a more comprehensive understanding.

## 1. Introduction

The circumpolar region, also known as the subarctic zone, is situated in the Northern Hemisphere between 50′ and 70′ NL. It encompasses the Alaska, Scandinavian, and Kola Peninsulas, most of Siberia, Canada, and the northern part of Scotland. Due to their high latitude, these areas experience extreme light conditions between summer and winter. During mid-summer, the subarctic regions undergo an all-night period of astronomical twilight without experiencing true night, as the sun never dips 18 degrees below the horizon [1]. This applies to areas located south of 66°34′ south latitude. The population residing in high latitudes is continually exposed to several factors, including prolonged low air temperatures, seasonal variations in light–dark cycles, and a harsh aerodynamic environment characterized by abrupt changes in barometric pressure [2,3]. Numerous studies have demonstrated that environmental factors and lifestyle significantly impact human adaptation in the Arctic. Common effects of climatic stress include reactions within the central nervous and endocrine systems, disruptions in sleep patterns, metabolic changes, and the onset of “oxidative stress”. Seasonal fluctuations in physiological processes and behavior have been observed in individuals living in modern society [4]. Light serves as the primary signal for synchronizing the body’s internal circadian rhythms with external cycles of light and darkness [5,6]. Both a lack of light and restricted exposure to light can profoundly affect children’s physiology, particularly their sleep–wake cycle. This impact is especially noteworthy for children living in high latitudes.

Growing evidence supports the significance of sleep as a crucial physiological process for replenishing daily energy expenditures and enhancing memory [7]. The recommended duration of sleep varies across different age groups and reflects the physiological changes during development [8]. Insufficient sleep ranks among the most prevalent health concerns among children and adolescents worldwide [9]. Extensive global studies have consistently found that students tend to stay up late on schooldays and compensate for their sleep deficit on weekends [10,11]. This discrepancy is observed not only in urban areas but also among populations in small settlements in the North, a phenomenon often referred to as social jetlag [12]. Another factor that could impact sleep duration is place of residence. Walch and colleagues found that later sunset times were associated with longer sleep duration among people from more than 20 different countries located around the globe [13]. Moreover, Friborg proposed that latitude could affect sleep duration by the different exposure to sunlight depending on the geographic zone [14]. Another research from Brazil compared two cities (i.e., Natal at 5°47′ S and Sao Paulo at 23°32′ S) and demonstrated that longer sleep phase delays were related to the further distance that people lived from the equatorial line [15]. In a study involving 87 countries, Randler highlighted that both climate and latitude contribute to an individual’s chronotype [16], a finding supported by Tonetti et al. [17]. Conversely, Benedito-Silva and colleagues did not find a clear trend in chronotype associated with latitude as a function of the photoperiod [18]. Moreover, this difference is notably distinct in the western regions of corresponding time zones in countries such as Russia [19], Germany [16], and Turkey [20].

Numerous studies have indicated a connection between sleep loss and metabolic syndrome, obesity, and diabetes in children and adolescents [21,22]. For instance, Sayin reported that extensive use of media and gadgets among children aged 10–13 resulted in a shortened sleep duration of less than 9 h per day. This was associated with higher insulin and HOMA-IR levels and lower levels of high-density lipoprotein cholesterol (HDL-C) compared to subjects sleeping 9–10 h or more per day [21]. An analysis by Sluggett and colleagues using data from the Canadian Health Measures Survey revealed that children and adolescents with shorter sleep durations had higher odds of being overweight or obese [22]. Additionally, a study from the US showed that adolescents sleeping less than 6.6 h per night displayed insulin resistance with compensatory insulin secretion compared to those sleeping ≥ 6.6 h [23]. Moreover, inadequate sleep can adversely affect cognitive abilities and learning skills [24]. Several researchers have established associations between sleep disorders such as excessive daytime sleepiness and the risk of stroke and coronary heart disease [25,26,27]. According to the latest Russian epidemiology reports, diabetes mellitus type 1 and metabolic syndrome in children and adolescents have latitudinal clines, with the highest rate being registered in northern Russia [28]. In our study, two studied settlements belong to the same economic district in Russia but differ by distance. The town of Apatity is located above the Polar Circle and 420 km north of the town of Kem’, which is situated in Northern Karelia. Therefore, the primary study objective was to explore the relationship between sleep-related factors, light exposure, social cues, physical activity, and potential confounding variables among schoolchildren residing in the European Arctic region using validated self-reported questionnaires. Additionally, we aimed to assess sleep hygiene in this pilot study.

## 2. Materials and Methods

Our study was conducted in two settlements situated in the Arctic region: Apatity, in the Murmansk Region (67°34′ N 33°23′ E), and Kem’, in Northern Karelia (64°57′ N 34°34′ E), during April and May 2019. Throughout this period, the duration of daylight was from 15.5 to 16 h. Participants for this study were recruited by the probability sampling method. First, we sent notices to all schools located in North-West Russia, obtained feedback from them, and randomly selected 3 schools in municipalities from the Murmansk region and 2 schools from Northern Karelia. Next, we randomly selected classes from the previously selected schools. Later, we selected a subsample consisting of Russian adolescents aged 13 to 15 years old (mean [SD] age: 14.34 [1.36] years; 40.4% boys) to run this pilot study. In our pilot study, we used propensity score matching with exact matching by sex and age among children living before and above the Polar Circle. The subjects were selected as part of a larger sample from our framework project, “Sleep Health in Russian Arctic”, as the non-profit volunteer project granted to S.N.K. School lessons commenced at 8:30 a.m. and finished between 1:30 p.m. and 2:15 p.m. in all assessed locations. According to the Russian educational system, regional schools could determine their schedule as either a five-day or six-day school week.

The inclusion criteria required acceptable educational skills in literacy (Russian language and mathematics) and children between 13 and 15 years old. All participants and their parents received preliminary information about the study’s objectives before enrollment. Written informed consent was obtained from the parent or legal guardian before the survey was administered. Children completed self-rating questionnaires individually in their classroom during an official school lesson. To secure data, all scholars put their completed questionnaires directly to the researcher; thus, neither classmates nor teachers were able to know the participants’ answers. Subsequently, children provided information regarding various sleep patterns, habits, lifestyle factors, and academic performance. We assessed all sleep disorders and behavioral patterns associated with sleep. Medical history, previous treatment, school performance, and validated questionnaires such as the Insomnia Severity Index (hereafter—ISI), and the Pediatric Daytime Sleepiness Scale (hereafter—PDSS) were evaluated. In summary, the validated Russian versions of PDSS, ISI, and the Munich ChronoType Questionnaire (MCTQ) were distributed in paper form to the participants. This study was approved by the Republican Medical Ethics Committee (Karelian Health Development and Petrozavodsk State University) under Protocol No. 41, dated 6 March 2018. This study was conducted following the ethical principles outlined in the Declaration of Helsinki and its subsequent amendments.

MCTQ: The Russian version of the MCTQ approved previously by Till Roenneberg and successfully applied by Russian researchers was used in our work [19,29,30]. Based on the MCTQ self-reported sleep behaviors, parameters were calculated on both schooldays and free days, including social jetlag, sleep duration, and the midpoint of sleep, which is an operationalization of chronotypes [15]. Chronotypes are given by midsleep on free days that have been corrected (MSFc), which represents a phase of the sleep–wake cycle. MSFsc is the corrected midpoint of sleep, accounting for weekend oversleep, i.e., it differs from the simple midpoint of sleep on free days. For details of the calculation, see work by Roenneberg et al. [29]. MCTQ parameters are expressed in hours, ranging from 0 h (extreme and morningness) to 12 h (extreme eveningness). We processed the completed surveys as previously described [19,30].

The MCTQ provided answers to the questions of times of falling asleep and awakening on work days and free days and of the use of an alarm clock on work days and free days. This information was used to calculate midsleep on free days that are sleep corrected (MSF_SC_), social jetlag (SJL), sleep duration on work days (SDw), and sleep duration on free days (SDf), as previously described [30]. MSFsc (chronotype estimate, cleaned of the confounder sleep debt) was calculated based on SDw/SDf, sleep onset on work days and free days (SOw/SOf), and midsleep on work days or free days (MSw/MSf), as follows: if SDf ≤ SDw: MSFsc = MSf = SOf + (SDf/2); if SDf > SDw: MSFsc = MSF − ((SDf − SDweek)/2) = SOf + SDweek/2, where SDweek = (5 × SDw + 2 × SDf)/7 for a 5-day work schedule (TR), and SDweek = (6 × SDw + SDf)/7, for a 6-day work schedule (ASW). MSFsc was calculated after excluding individuals who continued using an alarm clock on free days (n = 38/27 in TR/ASW were excluded, respectively). The social jet lag was calculated as MSf − MSw. We used the absolute value of SJL (|MSf − MSw|) but also discriminated between three subgroups: positive SJL (SJL+): SJL > 0; no SJL: SJL = 0; and negative SJL (SJL−): SJL < 0 in ASW and TR. Outdoor light exposure (OLE): OLE on work (OLEw) or free (OLEf) days was also considered.

PDSS: The Pediatric Daytime Sleepiness Scale (PDSS) is a self-estimated survey that consists of 8 items and assesses excessive daytime sleepiness in children and adolescents from 7 to 17 years old [31]. We previously validated the PDSS by test–retest, exploratory and confirmatory factor analyses, and Cronbach’s alpha for a Russian sample of children and adolescents [32]. The 5-point Likert scale spans from 0 (never) to 4 (always). A total score is calculated by summing up all the items to obtain values from 0 to 32.

The insomnia severity index (ISI) is developed to assess insomnia in adults and is commonly used in adolescents. The ISI represents a 7-item self-assessment tool describing the origin, severity, and impact of insomnia [33]. The 5-point Likert scale is applied to rate each item from 0 (no insomnia) to 4 (very severe problem), giving a total score ranging from 0 to 28. One should interpret the total score as follows: absence of insomnia (0–7), sub-threshold insomnia (8–14), moderate insomnia (15–21), and severe insomnia (22–28), respectively. This tool was initially translated into Russian by Konstantin Danilenko in 2011, who received consent to translate the questionnaire. Later, the Russian version of the questionnaire was validated by Elena Rasskazova [34].

Adolescent Sleep Hygiene Scale (AdSHS): The Adolescent Sleep Hygiene Scale (ASHS) is a self-reported tool addressed to evaluate sleep hygiene behaviors considered to affect the sleep quality and quantity of young children from the age of 12 years old, as follows [35]: physiological (e.g., evening caffeine use); cognitive (e.g., thinking about things that need to be completed at bedtime); emotional (e.g., going to bed feeling upset); sleep environment (e.g., falling asleep with the lights on); sleep stability (e.g., different bedtime/wake-time patterns on weekdays and weekends); substance use (e.g., evening alcohol use); daytime sleep (e.g., napping); and having a bedtime routine [35]. Although this tool was previously rated “approaching well established” in terms of its evidence-based assessment criteria, further investigation of the psychometric properties of the ASHS is needed, especially with older adolescents. Moreover, exploring connections of each sleep hygiene domain with measures of sleep quality and quantity will help to evaluate sleep hygiene recommendations. The Adolescents Sleep Hygiene Scale was translated into Russian and the authors obtained approval to translate ASHS and validate it into Russian from Monique LeBourgeois, who developed it.

Statistical data analyses: The research results were processed using the SPSS software version 22.0 from IBM Corporation (IBM, Armonk, NY, USA). Quantitative variables are presented as Median (Minimum; Maximum) in case of non-normal distribution, or mean (M) ± standard deviation (SD) in case of normal distribution. The normality of the variables was verified by the Shapiro–Wilk test. Linear regression and multiple regression models with co-factors were applied to assess the relationship between studied indicators. The χ^2^ test was used to test for differences between proportions.

## 3. Results

### 3.1. Cohort Characteristics

**Figure 1:**
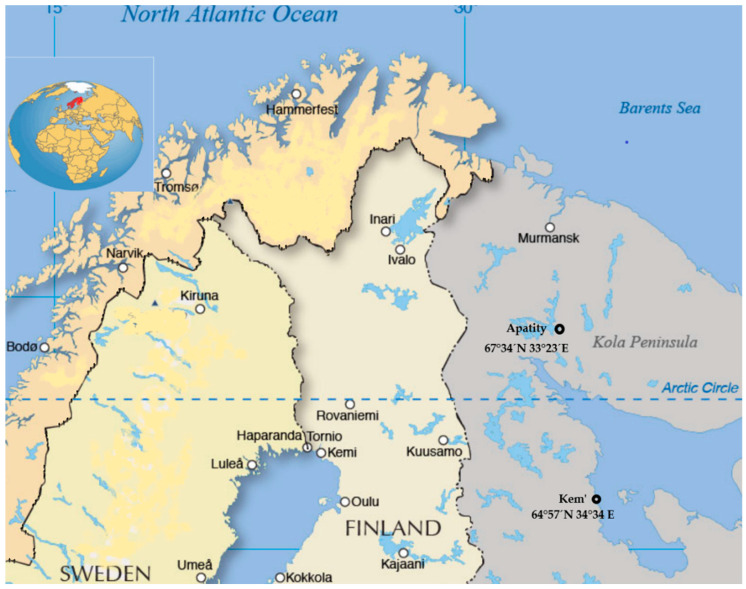
Geographic position of the studied settlements.

The main characteristics of our sample are presented in Table 1 and Table 2. We obtain experimental data from two Russian settlements lying closest in the European part to the Polar Circle—Kem’ and Apatity (Figure 1). One hundred participants were enrolled in the study with a response rate of 94%. In total, 6 participants, who did not fully complete the MCTQ, were eliminated, leaving 94 participants in the analysis sample. Of those, 56 (59.6%) were girls, and the mean age was 14.34 years old (Table 1 and Table 2).

No differences in anthropometric parameters were detected among the studied scholars (see Table 2). Several parameters showed significant differences including alcohol consumption, physical activity, and days at school. It should be noted that BMI values were in an acceptable range for adolescents in the total sample, as well as in both subgroups. However, Apatity scholars demonstrated a wider range of BMI scores than their colleagues from Kem’. Also, children from Apatity stayed at school more often and showed higher alcohol consumption than their counterparts from Kem’. It should be mentioned that Kem’ scholars were more physically active and achieved higher academic scores than those from Apatity (see Table 2).

### 3.2. Sleep Parameters on Schooldays and on Weekends

Data analysis of sleep variables revealed interesting findings for some sleep parameters between the groups (Table 3). First, bedtime on schooldays demonstrated a significant gap in sleep duration on schooldays between analyzed settlements. Most of the schoolchildren from both Apatity and Kem’ preferred to go to bed after 10 p.m., even on weekends (Table 3). That means scholars have a sleep shortage on schooldays and need to sleep more on weekends. Also, it explains the frequent use of the alarm clock to be awakened during the study week. This difference completely disappears on weekends. It could be explained also by sleep-onset times during schooldays. Of note, 26.3% of Kem’ scholars reported about an earlier bedtime. We expected a difference in sleep duration on schooldays between the analyzed groups. Indeed, it was less than the recommended range for teenagers aged 13–18 years old according to the American Academy of Sleep Medicine. Surprisingly, we did not detect any considerable difference in sleep duration between the two settlements. No one in Kem’ demonstrated a difference of more than 30 min in sleep duration on school and free days, compared to 100% of adolescents in Apatity (data not presented). Interestingly, more than 30% of Kem’ scholars reported a range for wake-up times before 7 a.m. and from 6 a.m. to 7 a.m. compared to 12.8% of those from Apatity (Table 3). Moreover, Kem’ scholars woke up before 7 a.m. on weekends (Table 3).

### 3.3. Sleep Complaints and Sleep Disorders in Schoolchildren

**Figure 2:**
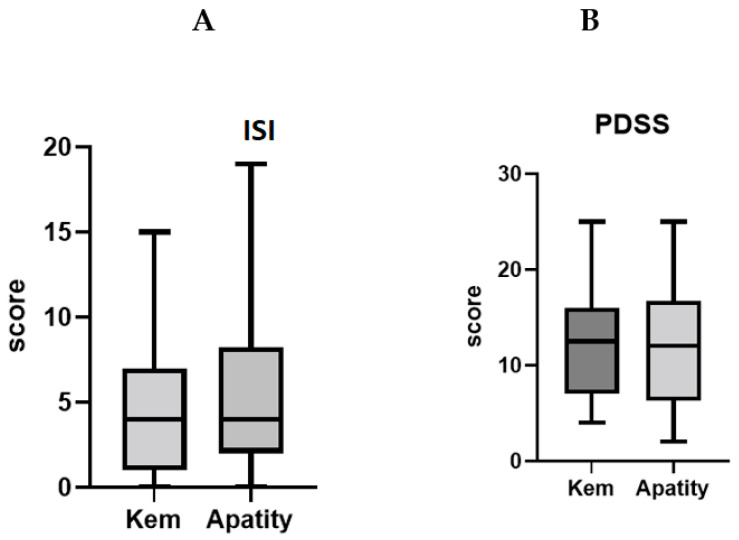
Comparison of insomnia symptoms severity and sleepiness in two study groups. (**A**) Insomnia Severity Index/Scale *p* = 0.56; (**B**) Pediatric Excessive Daytime Sleepiness *p* = 0.74.

To further elucidate the impact of sleep shortage, we studied the rate of sleep complaints, in particular, insomnia and excessive daytime sleepiness, in our sample. No difference between the study groups (Figure 2A) was found for either insomnia or excessive daytime sleepiness (Figure 2B). Moreover, moderate insomnia symptoms were reported in 18.4% of adolescents living in Kem’ and in 25% of respondents living in Apatity, respectively (data not presented).

Data analysis revealed no differences, either for individual questions or for the total PDSS scores. Mean PDSS values were reported for Kem’, 12.5 (4; 25), and Apatity, 12 (2; 25) (*p* = 0.74; Mann–Whitney test).

Excessive daytime sleepiness data revealed 48% of adolescents from both towns had values below the clinical threshold (PDSS ≥ 18) (Figure 2B). The overall rate of excessive daytime sleepiness in the sample was 17.1%. Next, the prevalence of excessive somnolence symptoms was reported in 19.6% of schoolchildren from Apatity and 13.2% for their coevals from Kem’ (*p* = 0.58).

**Figure 3:**
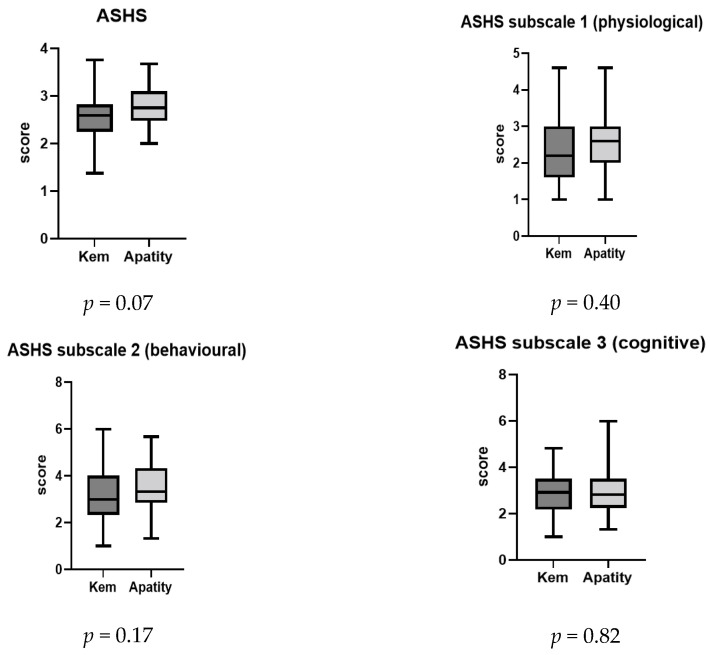

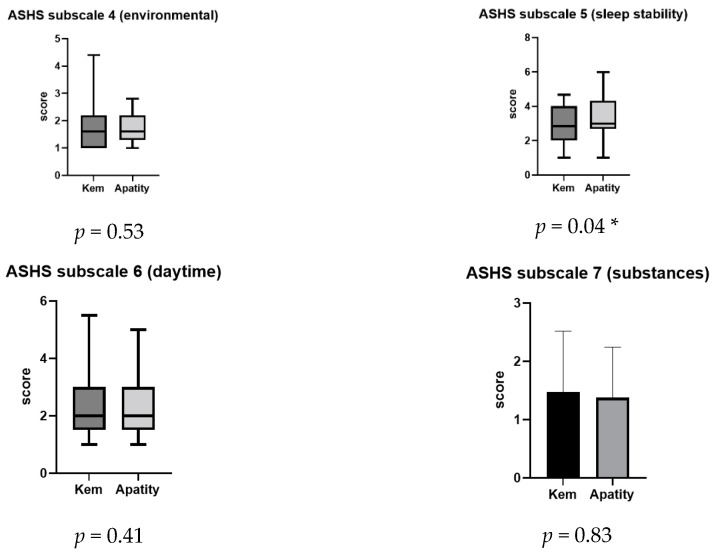
Comparison of sleep hygiene data based on ASHS in two study groups. Significant differences from Mann–Whitney rank-sum test are marked with an asterisk * *p* < 0.05.

Sleep hygiene scores were low in both investigated groups (see Figure 3). The average ASHS score was less than 4 in both samples, which indicates poor sleep hygiene (see also Table S1). A significant difference between Kem’ and Apatity subgroups was detected only for the sleep stability scale, with Kem’ scholars demonstrating lower values when compared to Apatity coevals.

### 3.4. The Association between Academic Achievements and Sleep Parameters

**Figure 4:**
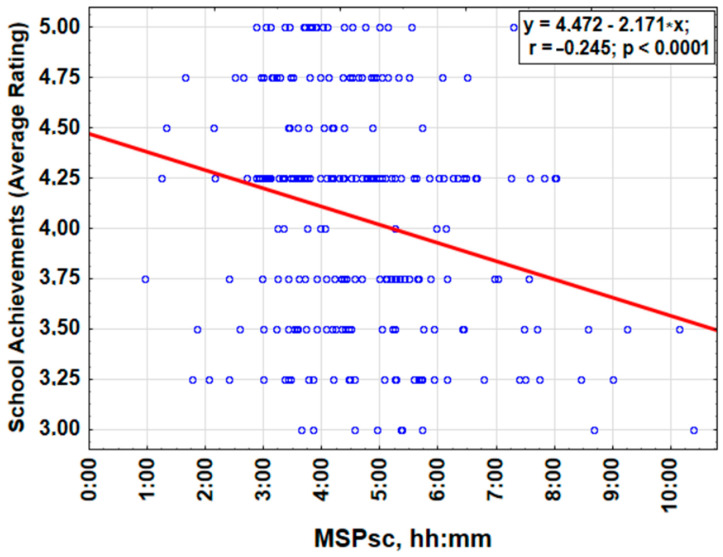
Correlation between academic scores (school achievements) and the sleep phase. Since the chronotype relates to both genders and outdoor light exposure, but also age, latitude, and alarm dependence, we performed multiple regression analysis considering MSPsc, age, OLEf, and OLEw, as co-dependent continuous predictors, and gender, latitude of residence, and alarm dependence on free days as categorical co-factors.

To find associations between sleep parameters, academic success, and demographic characteristics, we performed regression analysis (Figure 4 and Figure S1). Linear regression analysis has revealed few associations between some sleep parameters, academic success, and both ASHS and ISI scales. The most prominent correlations were between academic success and the difference in wake-up times between schooldays and weekends (min)—r = −0.339, *p* = 0.002—and between age and academic success—r = −0.225, *p* = 0.034. Moreover, other significant correlations were between age and the difference in wake-up times between schooldays and weekends: r = 0.274, *p* = 0.010. Also, one should note close but non-significant associations between PDSS scores and age (r = 0.196, *p* = 0.063) and between ASHS score and difference in wake-up times between schooldays and weekends (r = −0.223, *p* = 0.052), respectively.

Next, we decided to decipher the effect of outdoor light exposure on scholars in both studied locations. Linear regression analysis showed that school achievements were associated with gender (higher average rating in girls), chronotype (higher average rating in earlier MSPsc), and OLE on free days (higher average rating in “homebodies”). Multivariate regression analysis indicated that such parameters as gender, MSPsc, and OLEf were added to the model predicting school achievements (see Figure 4). 

## 4. Discussion

Studying the influence of extreme environmental factors on health in the population living in the Far North, as well as developing methods for the prevention and early diagnosis of diseases in children and adolescents, are priority tasks for pediatricians and health practitioners. Arctic and circumpolar regions represent the areas with specific climate conditions. Light deficiency in winter and its excess in summer have a harmful effect on the human body. This is essential for children and adolescents living in high latitudes. Our work explored self-reported sleep habits and the association between specified sleep parameters (sleep duration, chronotype, and outdoor light exposure) and academic success in 94 Russian adolescents between the ages of 13 and 15 years old.

Also, we investigated the impact of outdoor light on sleep parameters in schoolchildren living near the Polar Circle. Light is the main zeitgeber for the entrainment of intrinsic circadian rhythms to environmental changes [1,5,6,36]. In regular life, optimal light hygiene assumes sufficient time outdoors and sunlight exposure, which is commonly associated with relatively higher physical activity. These factors jointly maintain circadian robustness to benefit health and well-being [37,38,39]. Our study concurs with these principles, indicating that higher physical activity and longer sunlight exposure among Kem’ children on schooldays are associated with earlier wake-up times during schooldays, earlier bedtime whole week, reduced dependence on alarm clocks, and higher academic achievements. Pallesen reported on the same findings from Norway using the Cross-national Health Survey continued from 1983 to 2005 [40]. A Finnish study performed on a large sample including 1,136,583 adolescents aged 11–18 years found a lower insomnia rate than reported in our sample [41].

Modern youth actively use gadgets that are sources of artificial light [42,43]. Artificial light in the evening and at night not only serves as a factor for sleep disturbance but can also have (especially during these hours) an increasing effect on blood pressure [44,45]. In addition, exposure to artificial light at night (light pollution) is associated with suppression of melatonin production and desynchronization of the circadian body clock. Light desynchronization is considered a risk factor for the development of many diseases, in particular, obesity [43,46,47], metabolic syndrome/diabetes [48,49], and hypertension [50]. Another interrelated factor is chronotype, which may influence daily blood pressure dynamics and the risk of hypertension [51,52].

Sleep hygiene was poor in both investigated groups living in the circumpolar region. Such low values of ASHS could be explained by the negative effect of the time when the study was performed. Usually, Russian schoolchildren have transfer exams at this time. Our data agree with the results from Indian adolescents. Gupta reported that sleep hygiene was inadequate among all adolescents [53]. Similar studies were performed in Finland [41] and Norway [40].

The mean scores of excessive daytime sleepiness between two subgroups of Russian schoolchildren were lower than the data published in the original version of the questionnaire [31], but consistent with the results obtained previously by our group [54,55], and in Germany [24], the USA [56], and Korea [57]. This difference was associated with the age of the analyzed sample. Moreover, it is worth noting the seasonal impact on our data. Previously, we published a paper on the seasonal dynamics of excessive daytime sleepiness in the Russian Arctic [55]. The general level of daytime sleepiness determined in our sample, as well as in both subgroups, was lower than the clinical threshold determined at 18% according to Yang [58]. The results of older schoolchildren differ from many works published previously in the USA, Argentina, and Japan [56,59,60], which could be explained by the season when the study was performed. Our data show that children in the primary grades of school experience less daytime sleepiness compared to older children. At the same time, the risk of sleepiness in adolescents increased, which in turn implies a later start time for school for adolescents [60,61,62]. Here, we observed a negative impact on school performance and sleep parameters in children living in high latitudes, namely in circumpolar regions.

Our study has several limitations. Firstly, the limited number of surveyed schoolchildren might be due to the low population density in the Russian Arctic, which is significantly influenced by a low birth rate. Secondly, this study focused only on one season (spring). Future research encompassing assessments across all four seasons will provide a more comprehensive understanding. Thirdly, the lack of objective tools (such as actimeters, thermochrons, etc.) to monitor sleep parameters restricted the precise evaluation of sleep disorders. Fourthly, the small sample size in our work leads to non-significant *p*-values and indicates low power. To overcome these issues, we are working on expanding our study to other schools in multiple regions located in the Arctic area. Currently, there are no studies on sleep hygiene and the insomnia severity index in different regions of Russia. Available data from our colleagues showed no differences in pediatric daytime sleepiness scores from 9 regions of Russia (3772 participants between 10 to 18 years of age) [63].

This work was financially supported by the Ministry of Science and Higher Education of the Russian Federation (Agreement No. 075-15-2020-901). The funding organization had no role in designing or conducting this research. The author(s) have no proprietary or commercial interest in any materials discussed in this article.

## 5. Conclusions

Our study suggests that adolescents living above the Polar Circle tend to have sleep problems, e.g., late sleep-onset times, higher excessive daytime sleepiness, and insomnia-related symptoms because of experiencing reduced exposure to natural light compared to their coevals residing below the Polar Circle. Moreover, a heavier school schedule for those scholars might explain limited physical activity and sleep problems.

## Figures and Tables

**Table 1:** The number, location, and gender composition of the interviewed persons.

No	Settlements	Geographic Coordinates	Population, Persons *	N, Persons	Girls	Boys
1	Kem’ (Northern Karelia)	64°57′ N 34°34′ E	11,000	38	24	14
2	Apatity (Murmansk Region)	67°34′ N 33°23′ E	55,000	56	32	24
	Total			94	56	38

Note. * Data were extracted from the latest Russian census held in October 2022.

**Table 2:** Subject characteristics.

Variable	N	Total Sample	Kem’, N = 38	Apatity, N = 56	*p*-Level/X^2^/Degrees of Freedom
Age, years	94	14.34 (13; 15)	14.26 (13; 15)	14.39 (13; 15)	*p* = 0.154
Sex, males/females, %males ^a^	94	38/56 (40.4%)	14/24 (36.8%)	24/32 (42.86%)	*p* = 0.34; X^2^ = 0.56 df = 1
Height, cm	76	165 (130; 190)	165 (130; 182)	165 (152; 190)	*p* = 0.39
Weight, kg	75	55 (26; 110)	52 (26; 87)	56.5 (35; 110)	*p* = 0.16
BMI, kg/m^2^	75	19.8 (13.2; 40.4)	20.1 (13.2; 29.1)	19.8 (14.4; 40.4)	*p* = 0.35
Physical activity, yes/no ^a^	93	73 (78%)	35 (92%)	38 (67.9%)	*p* = 0.008 **; X^2^ = 7.051 df = 1
Alcohol consumption ^c^ Never Rarely Once a week Every day	94	81 (87.1%) 8 (8.6%) 5 (4.3%) 0	35 (92.1%) 1 (2.6%) 2 (5.3%) 0	46 (82.14%) 7 (12.5%) 3 (5.36%) 0	*p* < 0.001 ** X^2^ = 11.91 df = 1
Smoking, yes/no	93	8 (8.5%)	5 (13.2%)	3 (5.4%)	*p* = 0.19
Number of study days per week, 5 or 6 ^b^	94	5 (40.4%) 6 (59.6%)	5	6	*p* < 0.001 **
Academic success, average score (1–5)	94	4.4 (3; 5)	4.4 (3; 5)	3.6 (3; 5)	*p* = 0.20

Notes. Values are mean (standard deviation) and *n* (%) for a continuous and categorical variable, respectively. BMI = body mass index. ^a^ *p*-value Pearson Chi-square test was used; ^b^ Mann-Whitney test was used; ^c^ Fischer exact test was used. Weight status was determined based on the World Health Organization’s age- and sex-specific BMI. Significant differences are marked with two asterisks (**) (for *p* < 0.01).

**Table 3:** Sleep characteristics of the schoolchildren in Kem’ and Apatity.

Variable	N	Total Sample	Kem’	Apatity	*p*-Value
Sleep latency on schooldays, min	89	15 (0; 300)	20 (2; 300)	15 (0; 90)	0.19
Sleep latency on the weekend, min	88	15 (0; 300)	15 (2; 300)	15 (0; 60)	0.20
Bedtime on weekend Before 10:00 p.m. After 10:00 p.m.	89	7 (7.9%) 82 (92.1%)	5 (13.2%) 33 (86.8%)	2 (3.9%) 49 (96.1%)	0.13
Bedtime on schooldays Before 10:00 p.m. 10:00–11:59 p.m. After 00:00 a.m.	89	16 (18.0%) 59 (66.2%) 14 (15.8%)	10 (26.3) 25 (65.8%) 3 (7.9%)	6 (11.8%) 34 (66.7%) 11 (21.6%)	0.024 * Χ^2^ = 4.11
Sleep duration on schooldays, h	85	7.58 ± 1.27	7.47 ± 1.38	7.66 ± 1.17	0.52
Sleep duration on the weekend, h	84	9.20 ± 1.97	9.08 ± 2.36	9.30 ± 1.61	0.96
Daylight exposure on schooldays, min	94	285 (0; 600)	300 (60; 600)	270 (0; 630)	0.070
Daylight exposure on the weekend, min	94	150 (5; 570)	120 (20; 360)	180 (5; 570)	0.49
Alarm on schooldays	94	61 (64.9%)	20 (52.6%)	41 (80.4%)	0.005 **; Χ^2^ = 7.782
Alarm on the weekend	94	6 (6.4%)	3 (7.9%)	3 (5.9%)	0.71;
Wake-up on schooldays Before 7:00 a.m. After 7:00 a.m.	85	18 (21.2%) 67 (78.8%)	12 (31.6%) 26 (68.4%)	6 (12.8%) 41 (87.2%)	0.035 * Χ^2^ = 4.45
Wake-up on weekends Before 7:00 a.m. After 7:00 a.m.	84	3 (3.6%) 81 (96.4%)	3 (7.9%) 35 (92.1%)	0 46 (100%)	0.089

Notes. Significant (*p* < 0.05) differences between groups according to Mann–Whitney test are marked with an asterisk (*) (*p* < 0.05) or two asterisks (**) (for *p* < 0.01).

## Data Availability

The data presented in the current study are available upon request from the corresponding author. The data are not publicly available due to privacy and ethical restrictions.

## Supplementary Materials

The following supporting information can be downloaded at:https://www.mdpi.com/article/10.3390/children11030279/s1, Figure S1: Impact of chronotype on academic scores; Table S1: Specific sleep patterns reported by children.
