# Supplementary material for: Comparative Analysis of Sleep Hygiene and Patterns among Adolescents in Two Russian Arctic Regions: A Pilot Study
Source: Children (Basel). 2024 Feb 24;11(3):279. doi: 10.3390/children11030279 (PMC10969395; doi:10.3390/children11030279)
Supplement: Supplementary file 1 [file children-11-00279-s001.zip › children-2762672-supplementary.pdf]

Supplementary Files

Table S1: Specific sleep patterns reported by children

Awakenings at night, n (%)	93	25 (26.6%)	11 (28.9%)	14 (25.5%)	p = 0.71; $\chi^2 = 0.14$
The need for noise isolation at night, n (%)	92	39 (41.5%)	15 (39.5%)	24 (44.4%)	p = 0.64; $\chi^2 = 0.23$
The need for complete darkness at night, n (%)	93	34 (36.2%)	13 (34.2%)	21 (38.2%)	p = 0.15; $\chi^2 = 0.70$
Being afraid while sleeping, n (%)	93	28 (29.8%)	12 (31.6%)	16 (29.1%)	p = 0.07; $\chi^2 = 0.80$
Unusual behavior during sleep, n (%)	93	30 (31.9%)	13 (38.2%)	17 (30.9%)	p = 0.51; $\chi^2 = 0.48$
BP instability, n (%)	93	8 (8.5%)	3 (7.9%)	5 (9.1%)	p = 0.41; $\chi^2 = 0.84$

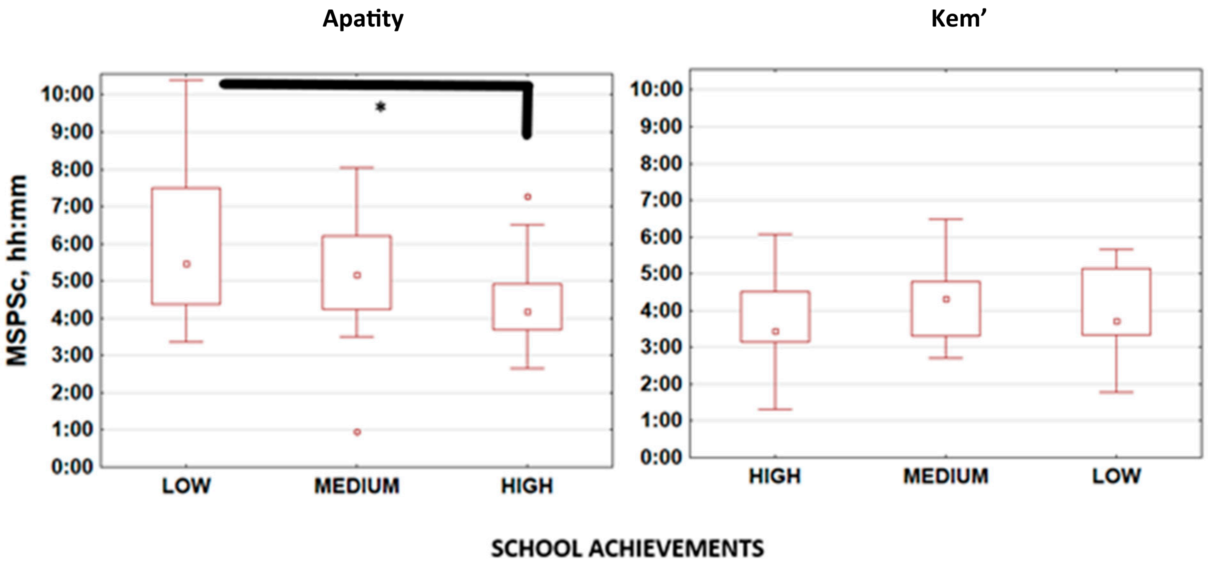

Figure S1. Impact of chronotype on academic scores. * $p < 0.05$ Tukey's HSD post hoc correction. Y axis reflects chronotype values expressed in local time . X axis means academic scores categorized by 3 groups as follows: High – 5; 5>4; Low – 3; 3>4; Medium – other academic scores. Russian educational system has the following rank scale:1-2 – bad; 3-4 low, 4 – good; 5-excellent.
